# Saccades Follow Perception When Judging Location

**DOI:** 10.1177/2041669515619513

**Published:** 2015-12-08

**Authors:** Funda Yildirim, Frans W. Cornelissen

**Affiliations:** Laboratory of Experimental Ophthalmology, University Medical Center Groningen, University of Groningen, Netherlands

**Keywords:** Eye movements, saccadic localization, perceived position, population encoding, decision making

## Abstract

An unresolved question in vision research is whether perceptual decision making and action are based on the same or on different neural representations. Here, we address this question for a straightforward task, the judgment of location. In our experiment, observers decided on the closer of two peripheral objects—situated on the horizontal meridian in opposite hemifields—and made a saccade to indicate their choice. Correct saccades landed close to the actual (physical) location of the target. However, in case of errors, saccades went in the direction of the more distant object, yet landed on a position approximating that of the closer one. Our finding supports the notion that perception and action-related decisions on object location rely on the same neural representation.

## Introduction

An ongoing debate in vision research concerns the question of whether perceptual decision making and eye movements (action) are based on different or shared neural representations. The possibility of different representations is suggested by current pathway theories of perception and action (Milner & Goodale, 2008). A related question is whether eye movements are directed toward the physical or perceived positions of objects (Schütz, Braun, & Gegenfurtner, 2011; Spering & Carrasco, 2015). The question has been addressed rather extensively for smooth pursuit eye movements, but only few studies have studied saccadic behavior.

Here, we address the perceptual and saccadic judgment of object distance, which provides a very direct way to test both questions. The general notion is that eye movements are very sensitive, and therefore oculomotor responses can be faster and more accurate than perception (Spering & Carrasco, 2015). In the present experiment, we test this. We hypothesized that if perceptual decision making and action are based on different neural representations, the eyes might still accurately localize objects even in case of perceptual errors. In that case, saccades would land on the actual (physical) location of an object rather than on its (erroneously) perceived position. However, in case of shared representations, we would expect the eyes to always follow perception, also in case of perceptual errors. In that case, saccades should land on an object’s (erroneously) perceived position.

## Results

**Figure 1.**
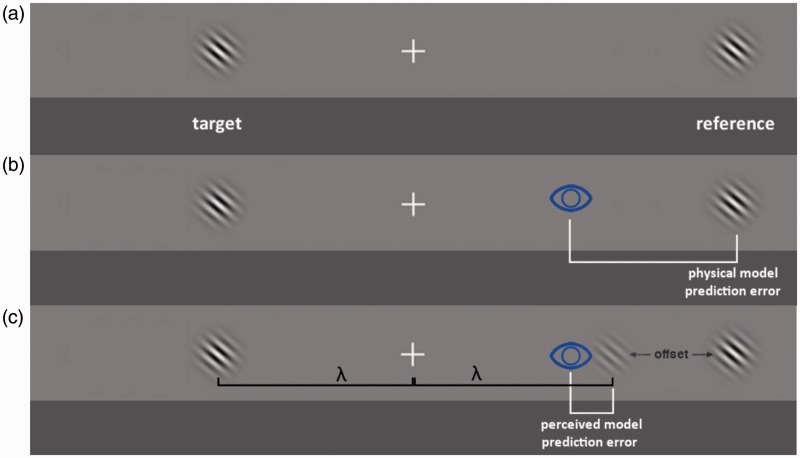
Panel (a). A stimulus consisted of two Gabor patches presented on the horizontal meridian, one to the left and one to the right of fixation. In a single trial, both patches were presented at either 8 or 10 degrees of eccentricity. One of the two objects received an eccentricity offset. The object physically closest to fixation was the target (here the left one) to which observers were instructed to make a saccade. Panel (b) Depiction of the physical model’s prediction error for erroneous responses (see main text for details). Eyes were intended to land on the reference. The prediction error is the distance between the eye’s landing position and the predicted landing position, that is, the position of the reference. Panel (c) Depiction of the perceived model’s prediction error (see main text for details). It assumes that erroneous responses arise because the reference is erroneously perceived to be the closer object. In this case, we thus assume that the saccade was intended to land at the mirrored target position (low-contrast Gabor, referred to as target placeholder). The prediction error is the distance between the saccade landing position and this placeholder position.

We performed an experiment in which observers decided on the closer of two objects—one presented in the left, the other in the right peripheral hemifield—to which they made a saccadic eye movement (Figure 1). From the saccadic responses, we derived two performance metrics.

**Figure 2.**
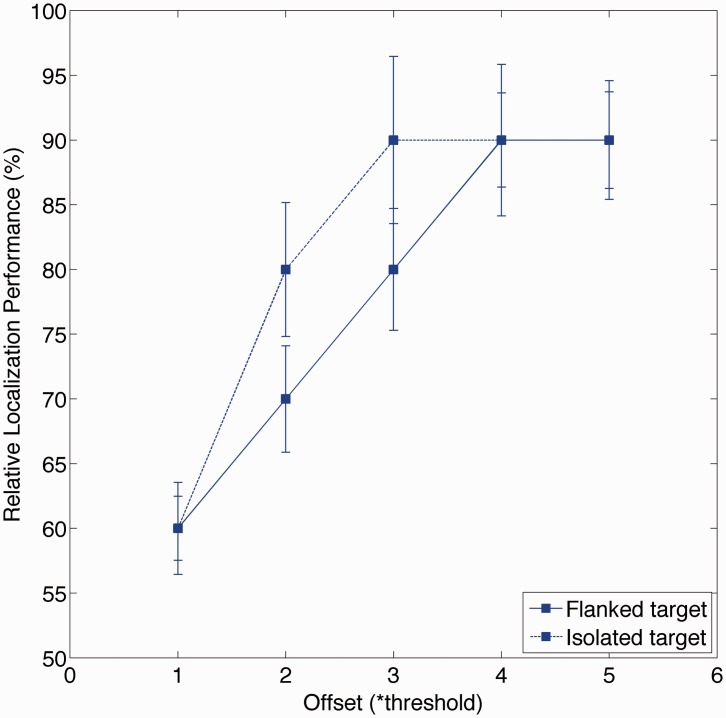
Relative localization performance. Symbols and bars indicate average ± saccadic eye movement of six observers. To enable averaging, offsets were expressed as multiples of the threshold offset required to achieve 80% correct relative localization performance for isolated objects. Bars indicate standard error of the mean over observers (Threshold: average ± *SE*: 0.5 ± 0.2 deg).

First, relative localization performance was defined as the percentage of trials in which the eyes moved in the direction of the target. Figure 2 shows that relative localization performance increased monotonously as a function of offset (the difference in position between the target and the reference). Of the trials with flankers, in about half of the trials, the flankers surrounded the target, and in the remaining trials, they surrounded the reference. This achieved the goal of increasing the number of errors for the smaller offsets (as expected based on the basis of crowding; Yildirim, Meyer, & Cornelissen, 2015).

To analyze absolute localization performance (i.e., the difference between the saccadic landing position and the target location), we postulated two models to predict saccadic landing position: 1. *Physical model*: The eyes were intended to land on the physical object (be it target or reference). 2. *Perceived model*: The eyes follow perception and were intended to land on the object perceived to be the closer one. In the case of correct trials, this predicts the same as Model 1. However, for erroneous trials, this model predicts that the saccade—while going in the direction of reference—was intended to land on or near the mirrored (virtual) position of the target (referred to as the target placeholder in Figure 1).

**Figure 3.**
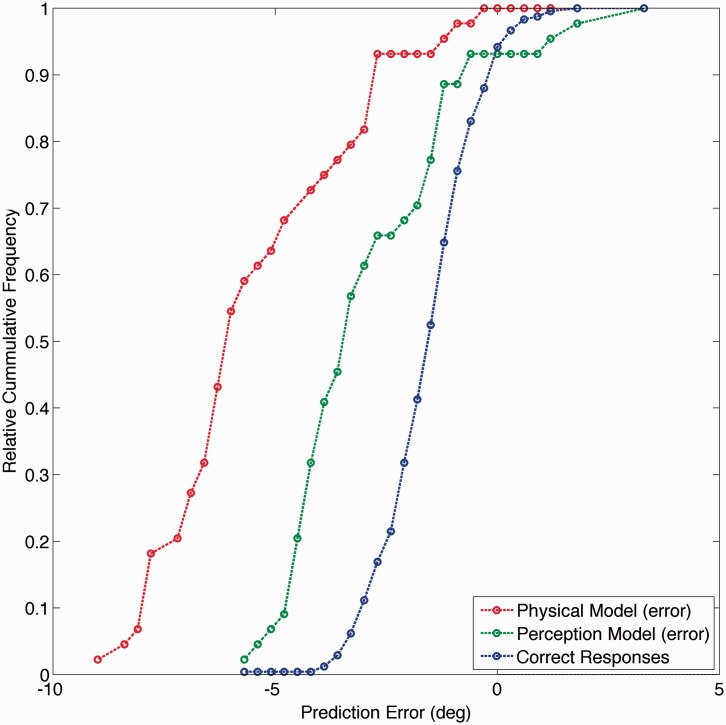
Cumulative distribution of errors in predicting saccadic landing position. Errors are shown for correct (red curve) and for erroneous responses (the latter for both the physical [blue] and perceptual [green] models). Data for a single representative observer for offset bins 4 and 5, and combining responses made in trials with and without flankers present.

For both models, we calculated the prediction error (i.e., the difference between the actual and the predicted saccadic landing position) on each trial. Figure 3 shows the data for a representative observer. Prediction errors are smallest for correct responses, but as indicated earlier, these do not discriminate between the two models. For erroneous responses, the perception model results in smaller prediction errors overall, compared with the physical model.

**Figure 4.**
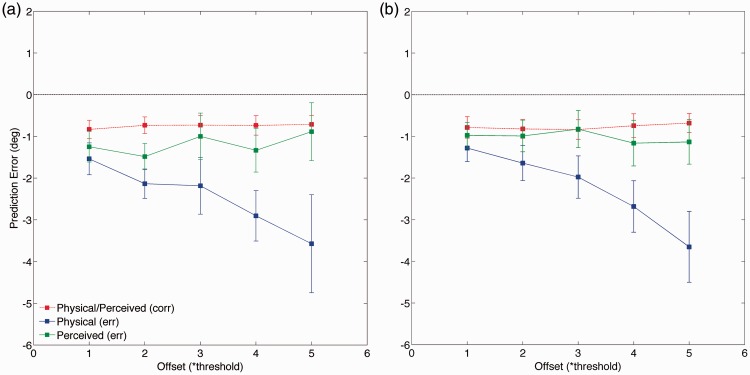
Prediction errors for saccadic landing position. Errors are shown as a function of eccentricity offset for the two models (indicated by symbols) predicting saccadic landing position for correct (dotted lines) and erroneous (filled lines) trials. Bars indicate standard error of the mean over observers. Panel (a): isolated objects. Panel (b): flanked objects. A within-subject repeated-measures ANOVA confirmed a significant interaction between model and offset for the erroneous responses (isolated: *F*(4, 20) = 15.9, *p* < .01; flanked: *F*(4, 20) = 35.7, *p* < .01). ANOVA = analysis of variance.

The average prediction errors are shown in Figure 4. In case of a good model, we expect a constant error, independent of the offset between target and reference positions. (Possibly, one may expect a deviation from zero because of a fixed saccadic undershoot.) On the other hand, for a bad model, we expect an increase or decrease in error with increasing offset. Both the physical and perceived models make (identical) good predictions for correct trials. However, for incorrect trials, the perceived model provides the best prediction. The prediction errors for saccades made in the conditions with or without flankers follow a highly similar pattern (compare Figure 4(a) and (b)). Accounting for saccadic undershoot did not influence the pattern of results. Neither did selecting the data based on either slower or faster than average saccadic responses.

## Discussion

The fact that the perceived model best predicted landing position in case of errors implies that saccade planning and perceptual decision making rely on the same neural representation. Our findings that saccades follow perception contrast the prevailing idea of separate pathways for perception and action (Milner & Goodale, 1992) and is more in line with the notion that the brain functions as a network (de Haan & Cowey, 2011).

Our results corroborate the finding that perceptual and saccadic decisions on object identity use identical information, suggesting that these too are based on similar representations (Bruno, Knox, & de Grave, 2010; Eckstein, Beutter, Pham, Shimozaki, & Stone, 2007). They are also in line with findings that saccadic localization is affected by motion shifting the perceived position of targets (Kosovicheva, Wolfe, & Whitney, 2014). However, this effect appears to depend on saccadic latency (de’Sperati & Baud-Bovy, 2008), whereas we did not find that this affected our results. On the other hand, our results contrast those of Kuhn & Land (2006) who, studying a vanishing ball illusion, found that observers did not look at the area where they claimed to have seen the ball vanish. However, the illusion studied is based on social cues, suggesting it relies on higher order representations rather than on actual object location.

A recent article (Lisi & Cavanagh, 2015) reported a dissociation between perception and action in a task that either required aligning the trajectories of moving peripheral targets or to make saccades to the end points of those same trajectories. They found that whereas perception of the trajectories was strongly influenced by motion inside the aperture of the target and thus deviated from the physical trajectories, action was seemingly directed at the physical location of the end points. These results clearly contrast our results that suggest that saccades are directed at the perceived, rather than the physical location of targets. A reason for these differential findings may be that our task was highly comparable for perception and saccades, whereas the perceptual and saccadic tasks of Lisi and Cavanagh’s study differed rather substantially (Morgan, 2015). This may have affected the outcome of their experiment, as the processing of spatial attributes such as motion and location is known to be highly dependent on the aspect that is emphasized by the task (J. Smeets, Brenner, de Grave, & Cuijpers, 2002; J. B. J. Smeets & Brenner, 1995).

### Limitations

In our current experiment, we did not record secondary saccades. Future experiments could consider this, as—among other things—it would allow to test the hypothesis that our results are at least partially the consequence of observers correctly locating the target and correctly programming saccadic amplitude, but assigning the saccade the wrong direction. This hypothesis, however, assumes that saccade amplitude and direction are programmed independently, a thesis for which there is only scarce evidence (Allik, Toom, & Luuk, 2003). Moreover, the prediction errors of the perceived model for erroneous responses do not completely overlap with those for correct responses, which would be expected if this were the primary explanation.

In the current—admittedly simple—perception model, we assume that the primary cause of errors lies in that the reference is wrongly perceived at the target location. A more extensive model could incorporate perceptual uncertainty for both target and reference positions (e.g., van den Berg, Johnson, Martinez Anton, Schepers, & Cornelissen, 2012) as well as uncertainty in saccadic programming.

While the flankers may have caused crowding of object and flankers, we cannot unambiguously draw a conclusion about the role of crowding, as we did not manipulate target-flanker similarity. Future experiments could take this aspect into account. Moreover, in the flanked condition, due to the averaging caused by crowding (Parkes, Lund, Angelucci, Solomon, & Morgan, 2001), the eyes’ landing position may have been influenced by flanker position. Unfortunately, we did not record assigned flanker positions, so this could not be verified. Finally, we presented the objects in a relatively small number of similar spatiotopic locations and only on the horizontal meridian (8 and 10 degrees of eccentricity ± offset). Future studies might consider expanding this to more complex or naturalistic situations.

### Final Conclusion

The location judgments indicated by the perceptual judgments (i.e., expressed through the direction that the eyes moved in) and by the landing positions of the eyes are very similar, supporting the notion that saccadic and perceptual decision making rely on a shared representation.

## Methods

### Subjects

Six different subjects (age range 21–29; 4 men and 2 women) participated in the experiment. One of the observers was an author (FY), and all the other observers were naïve as to the purpose of the experiment. All observers had normal or corrected to normal vision. The experiment was carried out in accordance with the institutional and national regulations and legislation and with the World Medical Association Helsinki Declaration as revised in October 2008.

### Apparatus

The stimuli were presented on a 22-inch CRT screen (LaCie; model electron22blueIV) with a resolution of 1920 × 1440 pixels and a refresh rate of 75 Hz. The screen had a background luminance of 55.5 cd/m^2^. A remote eye tracker (EyeLink 1000) was used to track eye movements at 500 Hz. Participants were seated in front of the screen at a viewing distance of 60 cm and with their heads resting in a headstand.

### Stimuli

Experiment and stimuli were programmed in MATLAB using the Psychtoolbox (Brainard, 1997) and the Eyelink Toolbox extensions (Cornelissen, Peters, & Palmer, 2002). A stimulus consisted of two Gabor patches (width = 1.0°, spatial frequency 3.0 cycles/deg, 7% contrast) presented on the horizontal meridian, one to the left and one to the right of a central fixation cross. In a single trial, both patches were presented at either 8 or 10 degrees of eccentricity, with a small negative or positive offset given to the target (details later). Base target and reference tilt was set to 45°. In 70% of the trials, Gabor patch flankers surrounded either the target or the reference, and these were positioned on an invisible circle. The separation between the target/reference and the flankers was always the same and proportional to the eccentricity (15% of the eccentricity). This was done to increase the number of errors, in particular for the smaller offsets. Each one of the flankers was randomly positioned in a quarter field divided by vertical and horizontal limits where the target or reference is at the center. Flanker tilt was set around 0° (horizontal) and randomly varied between 0° and 10° in each trial. Flankers were always presented at 25% contrast.

### Procedure

In their first visit, observers first completed a 100-trial training block. These were threshold detection trials, in which the eccentricity offset was between 0.1° and 1°. The base position was at either 8° or 10°. The distribution of the offset was determined by fitting a logarithmic distribution around the base position with an upper limit of 1 degrees of eccentricity. Following that, we individually determined the distance of the target to the fixation point that enabled observers to achieve 80% correct recognition performance at each eccentricity. This threshold was used to bin the actual offset values as explained in the next section.

In their next three visits, we ran 12 blocks of 260 trials and one block of 180 trials to reach 3,300 trials. Prior to each block, calibration was performed using the built-in routines of the EyeLink. Stimulus presentation commenced with pressing a key on the keyboard. Following that, fixation was checked. In case of correct fixation, the stimulus was presented. It consisted of two Gabor patches presented on the horizontal meridian, one to the left and one to the right of fixation. In a single trial, both patches were presented at either 8 or 10 degrees of eccentricity. One of the two objects received an eccentricity offset (total range: 0.03°–4.0°, but individually adjusted based on observer’s threshold). The object physically closest to fixation was the target to which observers were instructed to make a saccade.

As soon as the observer started a saccade to an object, the stimulus—except for the fixation point—disappeared. After the response saccade, the fixation point turned either red (error) or green (correct) to provide feedback to the observer.

### Data Analysis

Saccades were determined using the EyeLink’s built-in analyses routines. Prior to entering the statistical analysis, eye movement responses were filtered based on saccadic amplitude, latency, and direction. Trials were removed (a) in which saccades were made within 150 ms or after 1500 ms following the start of the stimulus presentation, (b) in which saccadic direction differed more than 15° from horizontal (the direction of the target or reference), or (c) in which saccadic amplitude was less than 1/2 of the target or reference eccentricity. On average, this excluded about 11% of the eye-response trials. From the saccadic responses, we derived two performance metrics.

### Relative Localization Performance

This metric was defined as the percentage of trials in which the eyes moved in the direction of the target. To enable averaging over observers, individual offsets were binned, by expressing them as multiples of the threshold offset required to achieve 80% correct relative localization performance for isolated objects. For each observer, performance was determined for each offset-bin, separately for conditions with and without flankers. Next, at the second level, results were averaged over observers.

### Absolute Localization Performance

For each of the three models (described in the main body of the text), we calculated the prediction error as the difference between the predicted and actual saccadic landing position on each trial. For each observer, prediction errors were binned according to offset-bin, relative response (correct or error), and model (physical and perceived). Next, at the second level, results for different models, response, and offset-bin were averaged over observers.

### Statistics

Statistical significance of the reported differences was verified using two-way repeated-measures analysis of variance with within-subject factors model (physical, perceived), and offset (five levels).
